# Vaccination with M2e-Based Multiple Antigenic Peptides: Characterization of the B Cell Response and Protection Efficacy in Inbred and Outbred Mice

**DOI:** 10.1371/journal.pone.0028445

**Published:** 2011-12-13

**Authors:** Amaya I. Wolf, Krystyna Mozdzanowska, Katie L. Williams, David Singer, Monique Richter, Ralf Hoffmann, Andrew J. Caton, Laszlo Otvos, Jan Erikson

**Affiliations:** 1 The Wistar Institute, Philadelphia, Pennsylvania, United States of America; 2 Center for Biotechnology and Biomedicine, Institute of Bioanalytical Chemistry, Universität Leipzig, Leipzig, Germany; 3 Department of Biology, Temple University, Philadelphia, Pennsylvania, United States of America; University of Georgia, United States of America

## Abstract

**Background:**

The extracellular domain of the influenza A virus protein matrix protein 2 (M2e) is remarkably conserved between various human isolates and thus is a viable target antigen for a universal influenza vaccine. With the goal of inducing protection in multiple mouse haplotypes, M2e-based multiple antigenic peptides (M2e-MAP) were synthesized to contain promiscuous T helper determinants from the *Plasmodium falciparum* circumsporozoite protein, the hepatitis B virus antigen and the influenza virus hemagglutinin. Here, we investigated the nature of the M2e-MAP-induced B cell response in terms of the distribution of antibody (Ab) secreting cells (ASCs) and Ab isotypes, and tested the protective efficacy in various mouse strains.

**Methodology/Principal Findings:**

Immunization of BALB/c mice with M2e-MAPs together with potent adjuvants, CpG 1826 oligonucleotides (ODN) and cholera toxin (CT) elicited high M2e-specific serum Ab titers that protected mice against viral challenge. Subcutaneous (s.c.) and intranasal (i.n.) delivery of M2e-MAPs resulted in the induction of IgG in serum and airway secretions, however only i.n. immunization induced anti-M2e IgA ASCs locally in the lungs, correlating with M2-specific IgA in the bronchio-alveolar lavage (BAL). Interestingly, both routes of vaccination resulted in equal protection against viral challenge. Moreover, M2e-MAPs induced cross-reactive and protective responses to diverse M2e peptides and variant influenza viruses. However, in contrast to BALB/c mice, immunization of other inbred and outbred mouse strains did not induce protective Abs. This correlated with a defect in T cell but not B cell responsiveness to the M2e-MAPs.

**Conclusion/Significance:**

Anti-M2e Abs induced by M2e-MAPs are highly cross-reactive and can mediate protection to variant viruses. Although synthetic MAPs are promising designs for vaccines, future constructs will need to be optimized for use in the genetically heterogeneous human population.

## Introduction

B cell responses and the generation of protective Ab titers are key determinants for antiviral immunity and the basis for successful vaccination. Current influenza virus vaccines elicit strong Ab responses against the viral glycoproteins hemagglutinin (HA) and neuraminidase (NA) and mediate sterile protection against reinfections with similar viral strains. However, their efficacy is limited due to the frequent mutations in HA and NA and the ability of viral subtypes to reassort and thus escape immune protection. This is highlighted by the recent outbreaks of avian influenza, emerging new pandemic H1N1 influenza A viruses, as well as the need to continuously update influenza vaccines to match the circulating strains. There remains, therefore, an urgent need to develop vaccines that elicit cross-reactive and protective immunity by targeting conserved viral proteins.

A promising target for the generation of cross-reactive immunity to multiple different influenza A viruses is the highly conserved 24-amino-acid N-terminal extracellular domain of the influenza virus M2 protein, termed M2 ectodomain (M2e) [1], [2]. M2 forms a disulfide-linked homotetramer in its native form and is expressed abundantly on the surface of infected cells [3], [4], [5], [6]. Passive transfer of anti-M2e monoclonal Abs in mice results in restricted viral replication and protection from viral challenge [5], [7], [8], [9], [10], [11]. Even under immune pressure in severe combined immunodeficiency (SCID) mice treated with anti-M2e monoclonal Abs, only two viruses with mutant M2e sequences emerged [9], thus highlighting the conserved nature of the M2e domain.

Humans have low to undetectable titers of anti-M2e Abs in their serum indicating that natural infection and current vaccines do not induce significant levels of anti-M2e Abs [12], [13]. To increase M2e-specific humoral immunity, several vaccination strategies have been evaluated in mouse and ferret models. M2e-based peptides, in particular synthetic multiple antigenic peptides (MAPs) containing M2e-peptides in combination with defined T helper determinants have been shown to successfully induce anti-M2e immunity [14], [15], [16], [17], [18], [19], [20], [21], [22], [23], [24]. Administration of M2e-MAPs in conjunction with potent adjuvants such as CpG ODN and CT, or its derivate CTA1-DD, elicited significant M2e-specific Ab responses and protected mice from viral challenge. Other vaccination strategies targeting M2e have been reported including M2-encoding plasmid DNA [25], M2-expressing recombinant viruses or virus-like particles [26], [27], [28], [29], [30] and several fusion proteins that link M2e peptides to immunogenic proteins [16], [31], [32], [33], [34], [35] or TLR ligands [36]. Most work with M2e-vaccine candidates has been performed in BALB/c mice, but a few studies examined responses in other inbred and outbred mice, ferrets, pigs or monkeys with varying results in terms of induction of anti-M2e Ab titers and protection [24], [27], [30], [37], [38], [39]. Recent results from Phase I clinical trials have raised hopes for applicability in humans (for review see [40], [41]).

Here we report the construction of a modified design of a M2e-based MAP vaccine. We investigated the correlates of the productive B cell response including the distribution of M2e-specific ASCs after different routes of administration, the Ab isotypes and their specificity as well as the protective efficacy to challenge with diverse influenza A viruses in multiple mouse strains.

## Results

### Improved design and synthesis of M2e-based multi antigenic peptide (MAPs) with promiscuous T helper epitopes

**Figure 1:**
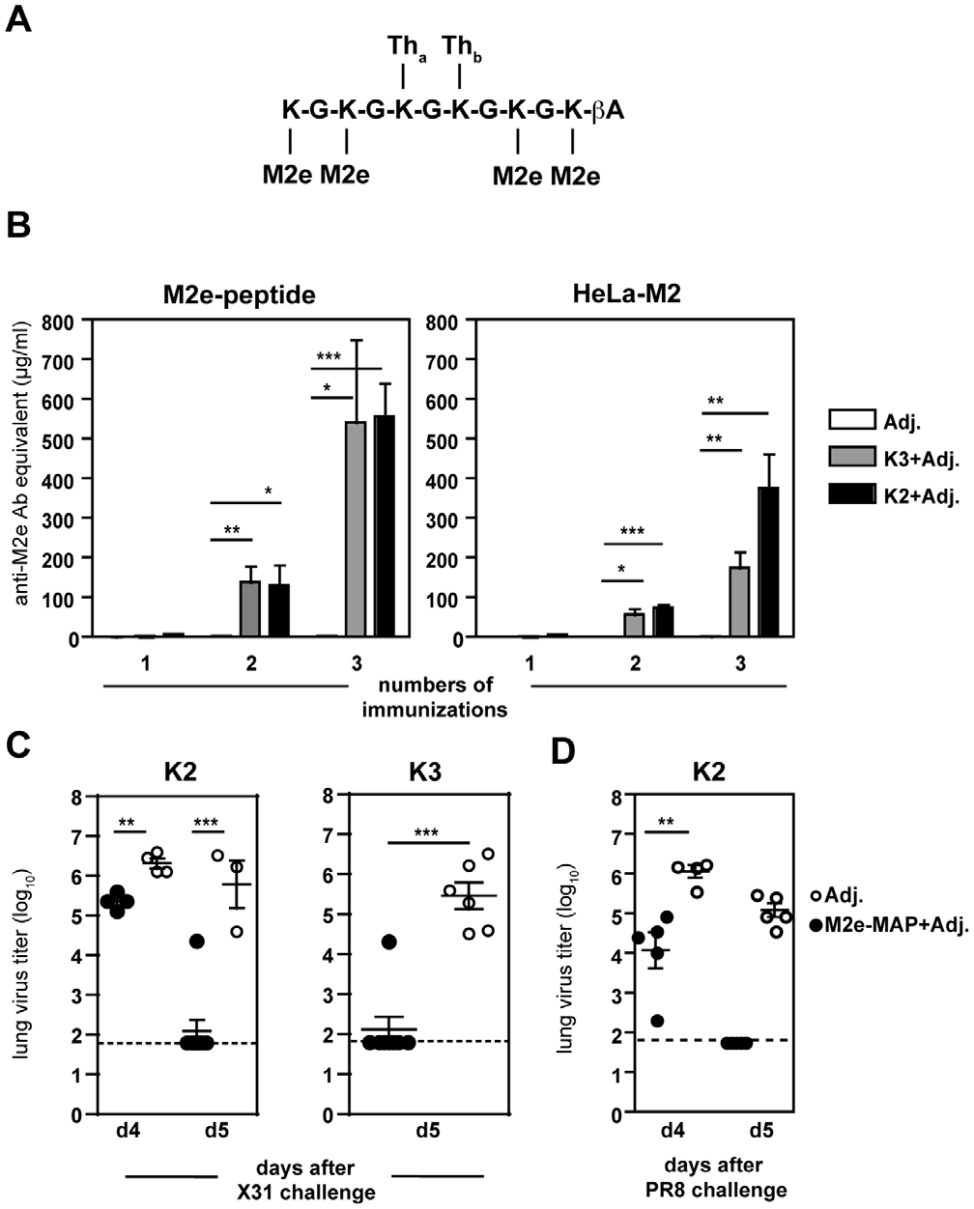
M2e-based multiple antigenic peptides induce high M2e-Ab titers and elicit protection against viral challenge. (**A**) Design of M2e-multiple antigenic peptides K2 and K3 depicting a branched construct of the peptide back-bone (lysine-glycine chain) with each four M2e residues and two T helper determinants (for sequences see **Table 1**). (**B–D**) BALB/c mice (n = 4–6/group) were immunized intranasally with M2e-MAP K2 or K3 mixed with adjuvants (Adj.) or adjuvants alone three times in 3–4 week intervals. (**B**) Sera were collected 3–4 weeks after the indicated number of immunization and measured in an ELISA against M2e-peptide or HeLa cells expressing full-length M2 (HeLa-M2). (**C and D**) 3–4 weeks after the third vaccination, mice were challenged with (**B**) 5 µL of 1000 TCID_50_ influenza virus A/X31 (2.5 µL/nare) or (**D**) 50 µL of 1000 TCID_50_ influenza virus A/PR8. Mice were euthanized 4 or 5 days after challenge as indicated and lungs assayed for infectious virus in MDCK cells.

The first-generation M2e-based multiple antigenic peptide, termed M2e-MAP G40d, contained four M2e side chains with the peptide sequence from influenza virus A/PR/8 (PR8) and two major PR8 HA T helper epitopes identified in BALB/c mice assembled on a polylysine-glycine scaffold peptide [14], [15]. It was shown to be highly immunogenic in BALB/c mice. With the aim to simplify the peptide synthesis and to create a construct that might be recognized by multiple haplotypes, new M2e-MAPs were generated. The new constructs were synthesized using an optimized orthogonal synthesis strategy compatible with conventional solid-phase synthesis that allowed for the generation of a single piece, multivalent and branched construct [42]. This not only facilitated the peptide synthesis in comparison to the original two-piece design that contained an oxidized cysteine-bridge but also made the final products chemically better defined [14], [15]. Two constructs were generated, termed K2 and K3, containing 4 M2e side chains corresponding to the 15 amino acid N-terminus of M2e from influenza virus PR8 and T helper epitopes identified as immunogenic peptide sequences from the circumsporozoite protein in *Plasmodium falciparum* (CS_381–396_ and CS_327–345_), the hepatitis B virus antigen (HBsAg_19–36_) and the hemagglutinin of influenza A virus (HA_307–319_) (see **Table 1**). These T helper cell epitopes are thought to be promiscuous as they can bind multiple MHC class II haplotypes in mice and humans [43], [44], [45], and therefore may induce M2e-specific immunity in a genetically diverse population. **Table 1** and **Figure 1A** illustrate the new M2e-MAP composition for K2 and K3 and lists the sequences of the peptides attached to the side-chains of the scaffold.

**Table 1:** M2e-based multiple antigenic peptides (M2e-MAPs).

	M2e epitope	Th_a_ determinant		Th_b_ determinant	
	*Sequence*	*Name*	*Sequence*	*Name*	*Sequence*
**K2**	SLLTEVETPIRNEWG	CS_381–396_	KKIAKMEKASSVFNVV	HBsAg_19–33_	FFLLTRILTIPQSLD
**K3**	SLLTEVETPIRNEWG	CS_327–345_	YLNKIQNSLSTEWSPCSVT	HA_307–319_	PKYVKQNTLKLAT

M2e-multiple antigenic peptides used in this study were termed K2 and K3. Constructs were synthesized to contain each four copies of the M2e epitope from influenza virus A/PR/8/34 and two T helper determinants, Th_a_ and Th_b_ (see **Figure 1A**). Names and corresponding amino acid sequences are listed.

### Induction of high M2e-specific Ab titers following immunization with M2e-MAPs in BALB/c mice

To test the ability of K2 and K3 to elicit anti-M2e Ab responses, BALB/c mice were vaccinated in a prime-boost regimen with 5 µg of M2e-MAPs in combination with adjuvants CpG 1826 and CT. Administration of M2e-MAP G40d in combination with these adjuvants induced protective serum anti-M2e Ab titers in BALB/c mice [14], [15]. We tested the specificity of the elicited Ab response by two means: first, binding to linear M2e-peptide, cys-M2e, and secondly binding to cell-surface-expressed native tetrameric M2 protein using a stably transfected cell line, HeLa-M2 [12]. As measured by both assays, K2 and K3 induced anti-M2e Abs after two intranasal (i.n.) immunizations that were amplified with an additional boost (**Figure 1B**). K2 and K3 induced greater anti-M2e serum Ab titers as compared to the original M2e-MAP G40d as previously shown [14], [15].

To address whether vaccination with K2 and K3 induced protection, mice were challenged with influenza virus A/HKx31 (X31) and assayed for infectious virus in their lungs four and five days later. On day 4 after challenge, K2-immunized mice had significantly reduced viral titers as compared to a cohort of mice that received adjuvants alone (**Figure 1C**). By day 5 after challenge, the majority of mice that received K2 or K3 in combination with adjuvants had undetectable level of virus in their lungs, whereas all mice in the control group exhibited high viral titers. Similar protection efficacy was also observed for the first-generation M2e-MAP G40d [14], [15]. Nevertheless, as we show here K2 and K3-immunized mice were protected even when challenged with the more pathogenic strain PR8 (**Figure 1D**).

### Characterization of the anti-M2e response to different routes of immunization with M2e-MAPs

**Figure 2:**
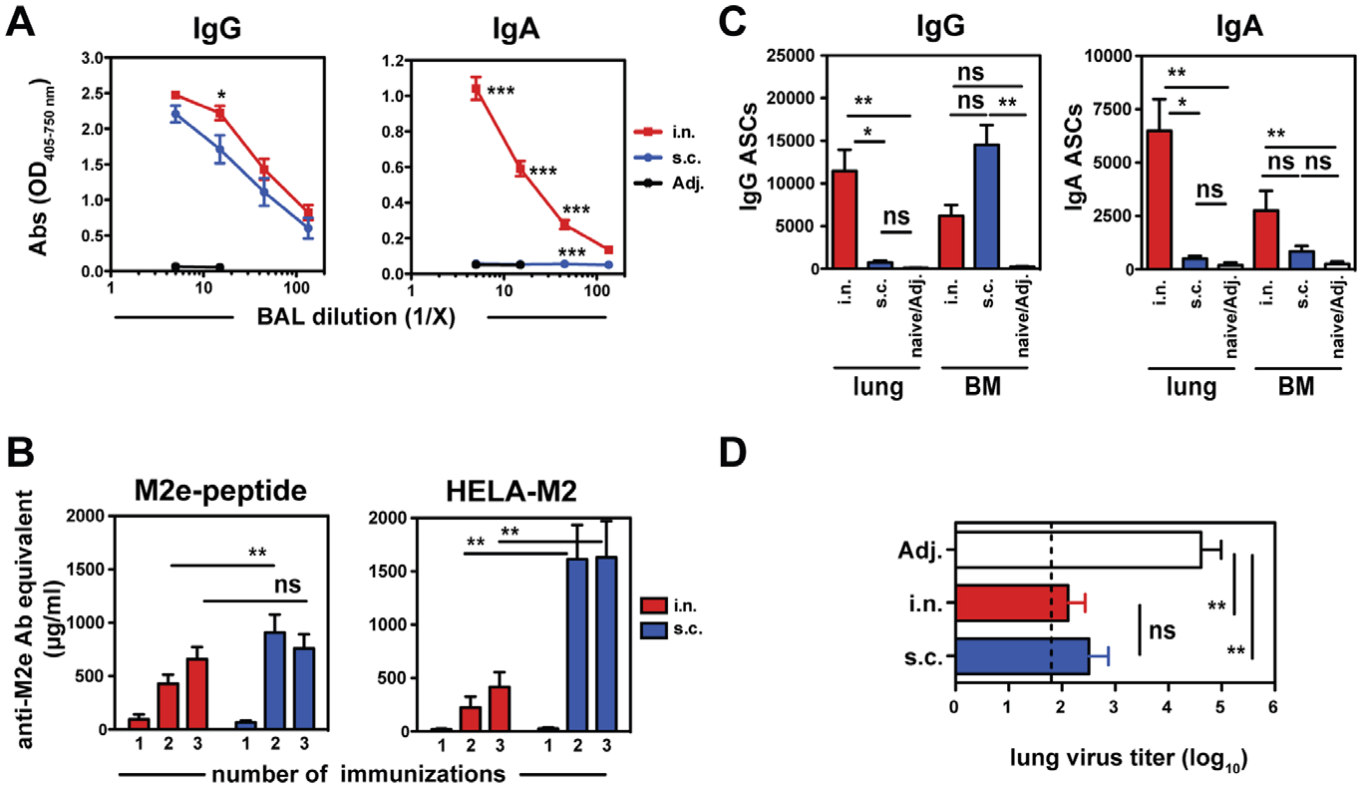
Route of immunization with M2e-MAPs determines systemic and respiratory tract responses. BALB/c mice were immunized with K2 and adjuvants or adjuvants alone either intranasally (i.n.) or into the tailbase (s.c.) as described above. (**A**) M2e-specific Abs in the bronchio-alveolar lavage (BAL) fluid after third vaccination were determined by ELISA on M2e-peptide (n = 3 mice/group). (**B**) Sera were collected 3–4 weeks after the indicated number of immunizations and measured by ELISA for binding to M2e-peptide and HeLa-M2 cells. (**C**) Mice were euthanized after third vaccination and cells from lungs and bone marrow (BM) seeded onto ELISPOT plates coated with M2e-peptide. Plates were developed with anti-mouse IgG or IgA. Results from 4–6 mice from 2 independent experiments are expressed as mean counts (± SEM). (**D**) Groups of mice were challenged with influenza virus A/X31 (1000 TCID_50_/5 µL) and infectious virus in the lungs determined 5 days after challenge.

Previous reports suggest that the nature of the protective anti-viral B cell response generated locally in the lungs differs depending on the route of immunization [14], [46]. To determine the impact of the route of vaccination on the level of Abs in the lungs versus Ab levels in the serum the M2e-specific Ab responses to K2 after i.n. and s.c. immunization were compared. Similar levels of IgG Abs against M2e were detected in the BAL fluid independent of the route of administration (**Figure 2A** and data not shown). S.c. vaccination induced significantly higher levels of M2e-specific IgG Abs in serum as measured by ELISA on M2e-peptide and HeLa-M2 cells (**Figure 2B**). M2e-specific IgA Abs were only detectable in the BAL fluid after i.n. but not s.c. immunization. M2e-specific IgA was undetectable in sera of vaccinated mice (data not shown and [14]).

To determine the origin of IgA in BAL after i.n. vaccination, we developed an enzyme-linked immunosorbent spot (ELISPOT) assay on plates coated with M2e-peptide to detect M2e-specific ASCs. ELISPOTs revealed higher numbers of IgA and IgG ASCs in the lungs after i.n. *versus* s.c. immunization (**Figure 2C**). IgG ASCs were predominantly induced with s.c. immunization and found in the BM, whereas IgA ASCs in the BM were higher after i.n. immunization. Similar results were observed with ELISPOTs on HeLa-M2 cells (data not shown).

To address whether the differences in the anti-M2e B cell response elicited by these distinct routes of immunizations had an impact on protection, mice were challenged with X31 virus and virus titers in the lungs determined 5 days later. BALB/c mice immunized i.n. or s.c. with K2 and adjuvants had 100-fold lower viral titers as compared to mice that received adjuvants alone, however there was no significant difference between the groups of i.n. or s.c. immunized mice (**Figure 2D**). Thus, while i.n. and s.c. immunization with K2 induced different Ab isotypes and ASC localization, mice were equally well protected against viral challenge.

### Cross-reactivity to viruses and peptides with variant M2e-sequences

**Figure 3:**
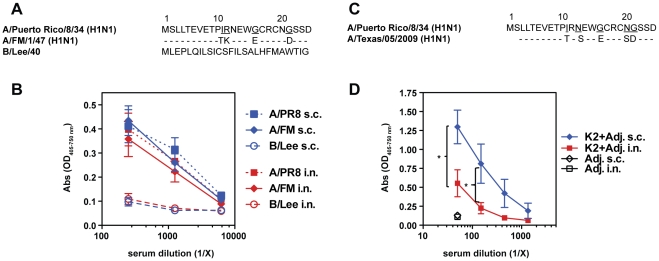
Serum Abs induced with M2e-MAP K2 exhibit cross-reactivity. (**A**) M2e amino acid sequence comparison of influenza virus A/PR8, A/FM and B/Lee. (**B**) Serum Abs of BALB/c mice (n = 5 mice) immunized i.n. or s.c. with K2 peptide and adjuvants were tested in an ELISA for binding to MDCK cells infected with influenza virus A/PR8 (square), A/FM (diamond) and B/Lee (circle). (**C**) M2e amino acid sequence from the pandemic influenza strain A/Texas/2009. (**D**) Sera with anti-M2e Abs induced by i.n. or s.c. immunization with K2 peptide and adjuvants or adjuvants alone were tested for binding to a synthetic peptide corresponding to the A/Texas/2009 M2e sequence as depicted in (**C**).

We tested whether immunization with K2 induced Abs able to recognize cells infected with viruses containing divergent M2e sequences. Sera from mice immunized i.n. or s.c. with K2 and adjuvants were assayed for binding to MDCK cells infected *in vitro* with the influenza A viruses PR8 and A/FM1/49 (FM) and an influenza B virus, B/Lee/40. As shown in **Figure 3A**, M2e from FM differs by 4 amino acids from PR8-M2e, while M2e from B/Lee is greater than 95% different from PR8. Equal binding was observed from serum Abs of mice immunized with K2 and adjuvants to cells infected with A/PR8 or A/FM and no binding was measured to B/Lee-infected cells (**Figure 3B**). There was no detectable difference between sera from i.n. or s.c. immunized mice (data not shown).

We extended our studies to test whether K2 induced serum Abs were able to cross-react with the M2e domain of the pandemic influenza strain A/Texas/2009 (pH1N1), which has 5-amino acid differences from the vaccine-encoded PR8-M2e sequence. Sera from mice immunized s.c. with K2 and adjuvants displayed significantly more binding than sera from i.n. immunized mice (**Figure 3D**), possibly due to higher anti-M2e IgG serum Ab levels as compared to i.n. immunized mice (**Figure 2B**). Our data suggest that immunization with K2 induces different degrees of cross-reactive Abs and that the degree of binding to specific M2e-target sequences depends on the route of vaccine administration.

### Protection against mutant M2e-escape viruses

**Figure 4:**
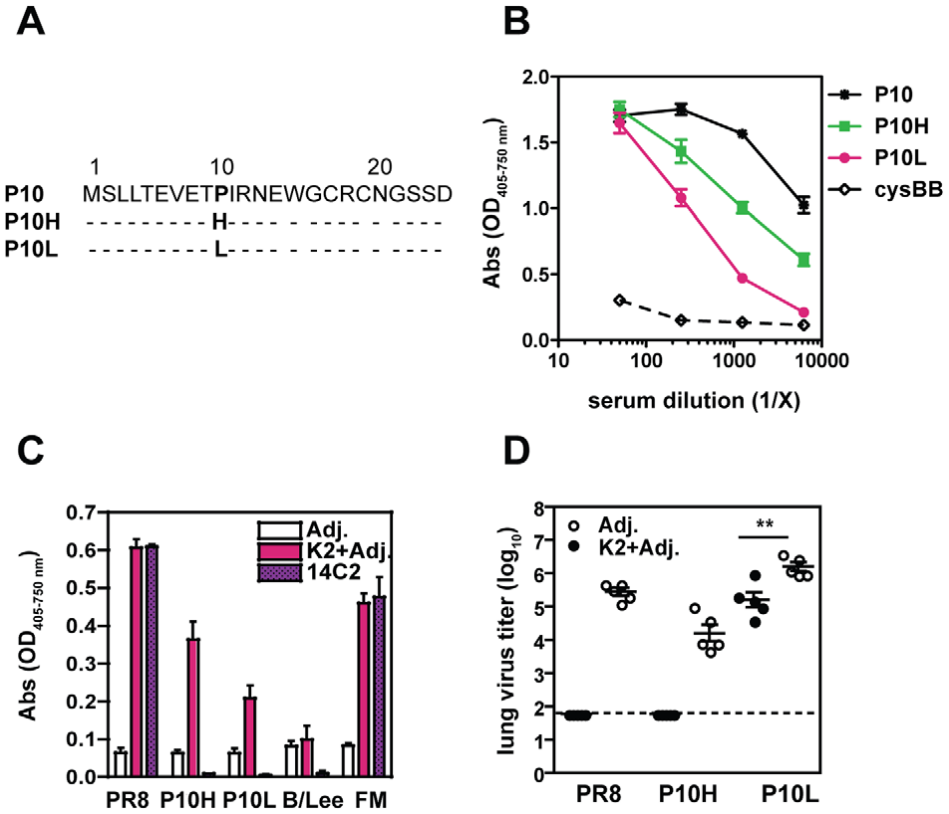
Immunization with M2e-MAP K2 protects against challenge with M2e-variant viruses. (**A**) Amino acid sequence comparison of M2e from influenza A/PR8 (P10) or M2e-escape mutant viruses (P10H and P10L) as described in Ref. 9. (**B**) Sera from BALB/c mice immunized i.n. three times with K2 peptide and adjuvants were assayed in an ELISA on peptides with M2e sequences as shown in (**A**) or control peptide cysBB: cysteine back-bone. (**C**) Pooled sera (1∶250 dilution) from BALB/c mice (n = 5 mice) immunized as in (**B**) were assayed in an ELISA for binding to MDCK cells infected with influenza virus A/PR/8, the M2e-escape mutants P10H and P10L, A/FM and B/Lee. The monoclonal anti-M2e Ab 14C2 (1 µg/mL) is shown as control. (**D**) BALB/c mice (n = 5 mice/group) immunized i.n. with K2 peptide and adjuvants were challenged with 1000 TCID_50_/50 µL influenza virus A/PR/8, P10H and P10L three weeks after the third vaccine administration. Infectious virus in the lungs was determined 5 days after challenge.

Treatment of PR8-infected SCID mice with monoclonal anti-M2e Abs resulted in the emergence of 2 viruses with mutations at amino acid position 10 [9]. The M2e-escape mutant virus designated P10H, has an amino acid change from proline to histidine at position 10, and the virus termed P10L (**Figure 4A**) has a proline to leucine exchange at position 10. To test whether Abs induced by i.n. administration of K2 recognized these mutations, sera from K2-immunized mice were tested in an ELISA against peptides corresponding to parental PR8 (P10) and mutant viruses. Serum Abs bound to all peptides, although binding to P10L and P10H was reduced with increasing serum dilution (**Figure 4B**). Likewise, serum Abs showed robust binding to MDCK cells infected *in vitro* with influenza virus A/PR/8, and to lesser degrees to cells infected with the mutant viruses P10H and P10L (**Figure 4C**). Similar results were obtained with sera from mice that were s.c. immunized (data not shown). The monoclonal anti-M2e Ab, 14C2, served as a positive control for binding to PR8 and FM, whereas it was unable to recognize MDCK cells infected with the escape mutants as shown previously [9].

To test whether the induction of cross-reactive Abs confers protection, K2-immunized mice were challenged with P10H, P10L and parental PR8 virus and infectious virus in the lungs determined 5 days later. None of the mice challenged with PR8 or P10H virus had detectable virus in the lungs at that time (**Figure 4D**). After challenge with P10L, K2-immunized mice showed a 10-fold reduction in P10L virus titers as compared to control cohorts. Together, these results suggest that immunization with K2 and adjuvants generates a pool of cross-reactive Abs able to protect against viruses with variant M2e-sequences.

### Responses in different inbred and outbred mouse strains

K2 and K3 M2e-MAP constructs contain promiscuous T helper epitopes and were synthesized with the goal to generate a vaccine able to be used in a genetically diverse population. To test whether the K2 and K3 peptide vaccines show efficacy in mouse strains other than BALB/c mice (H-2*^d^*), we extended our studies to C57BL/6 (H-*2^b^*) and C3H (H-*2^k^*) mice, as well as the outbred mouse strains CD1/ICR and Swiss Webster (SW). As summarized in **Table 2**, i.n immunization with K2 in combination with the adjuvants CpG 1826 and CT was able to elicit a significant anti-M2e Ab response in BALB/c, but not in C57BL/6, C3H, CD1/ICR and SW mice as measured by ELISA on both M2e-peptide and HeLa-M2 cells. Likewise, immunization of outbred CD1/ICR mice with K3 and adjuvants also failed to induce measurable anti-M2e Abs (data not shown). Moreover, when K2-immunized mice were challenged with X31 virus to test whether mice were protected from viral infection, only BALB/c mice had significantly reduced viral titers in their lungs and noses at day 5 after challenge (**Table 3**). Thus, the absence of protection observed in mouse strains other than BALB/c mice correlates with the failure to elicit anti-M2e-Abs in these mice following M2e-MAP immunization.

**Table 2:** Anti-M2e Ab induction in various mouse strains following vaccination.

M2e-assay	Mouse strains	Groups	
		Adjuvants	K2+Adjuvants
M2e-peptide	**BALB/c (I-A^d^, I-E^d^)**	2.2±2.3	**783.9±243.8**
	**C3H (I-A^k^, I-E^k^)**	<1	**1.0±0.3**
	**C57BL/6 (I-A^b^, I-E^o^)**	<1	**<1**
	**CD1/ICR**	1	**2.5**
	**Swiss Webster**	1	**2.5**
HeLa-M2	**BALB/c (I-A^d^, I-E^d^)**	2±1.9	**407.9±206.6**
	**C3H (I-A^k^, I-E^k^)**	<1	**<1**
	**C57BL/6 (I-A^b^, I-E^o^)**	<1	**<1**
	**CD1/ICR**	<1	**7.6**
	**Swiss Webster**	<1	**<1**

BALB/c (n = 21), C3H (n = 5), C57BL/6 (n = 3), CD1/ICR (n = 7) and Swiss Webster (n = 5) mice were immunized i.n. with adjuvants CpG 1826 and CT alone or K2 and adjuvants. After the 3^rd^ immunization sera were tested for M2e-specific Abs (in µg/mL) by ELISA on M2e-peptide and HeLa-M2 cells as indicated. Results are displayed as mean ± SD or results are shown from pooled samples.

**Table 3:** Viral titers in lungs and nose of X31-challenged mice following vaccination.

Virus titers	Mouse strains	Groups	
		Adjuvants	K2+Adjuvants
Lungs	**BALB/c (I-A^d^, I-E^d^)**	5.8±1.0	**2.0±0.7**
	**C3H (I-A^k^, I-E^k^)**	4.1±0.8	**4.1±1.6**
	**C57BL/6 (I-A^b^, I-E^o^)**	5.7±1.0	**4.8±0.6**
	**CD1/ICR**	5.1±0.3	**4.3±1.6**
	**Swiss Webster**	5.0±0.1	**4.9±0.7**
Nose	**BALB/c (I-A^d^, I-E^d^)**	4.8±0.3	**3.3±0.7**
	**C3H (I-A^k^, I-E^k^)**	4.8±0.2	**4.6±0.2**
	**C57BL/6 (I-A^b^, I-E^o^)**	4.8±0.1	**5.1±0.6**
	**CD1/ICR**	5.1±0.2	**5.1±0.3**
	**Swiss Webster**	5.2±0.6	**5.2±0.4**

BALB/c (n = 21), C3H (n = 5), C57BL/6 (n = 3), CD1/ICR (n = 7) and Swiss Webster (n = 5) mice were immunized three times i.n. with adjuvants CpG 1826 and CT alone or K2 and adjuvants and tested for their protection against viral challenge with X31 virus (1000 TCID_50_ in 5 µL). Virus titers in lungs and noses were determined 5 days after challenge. Results are displayed as mean ± SD.

**Figure 5:**
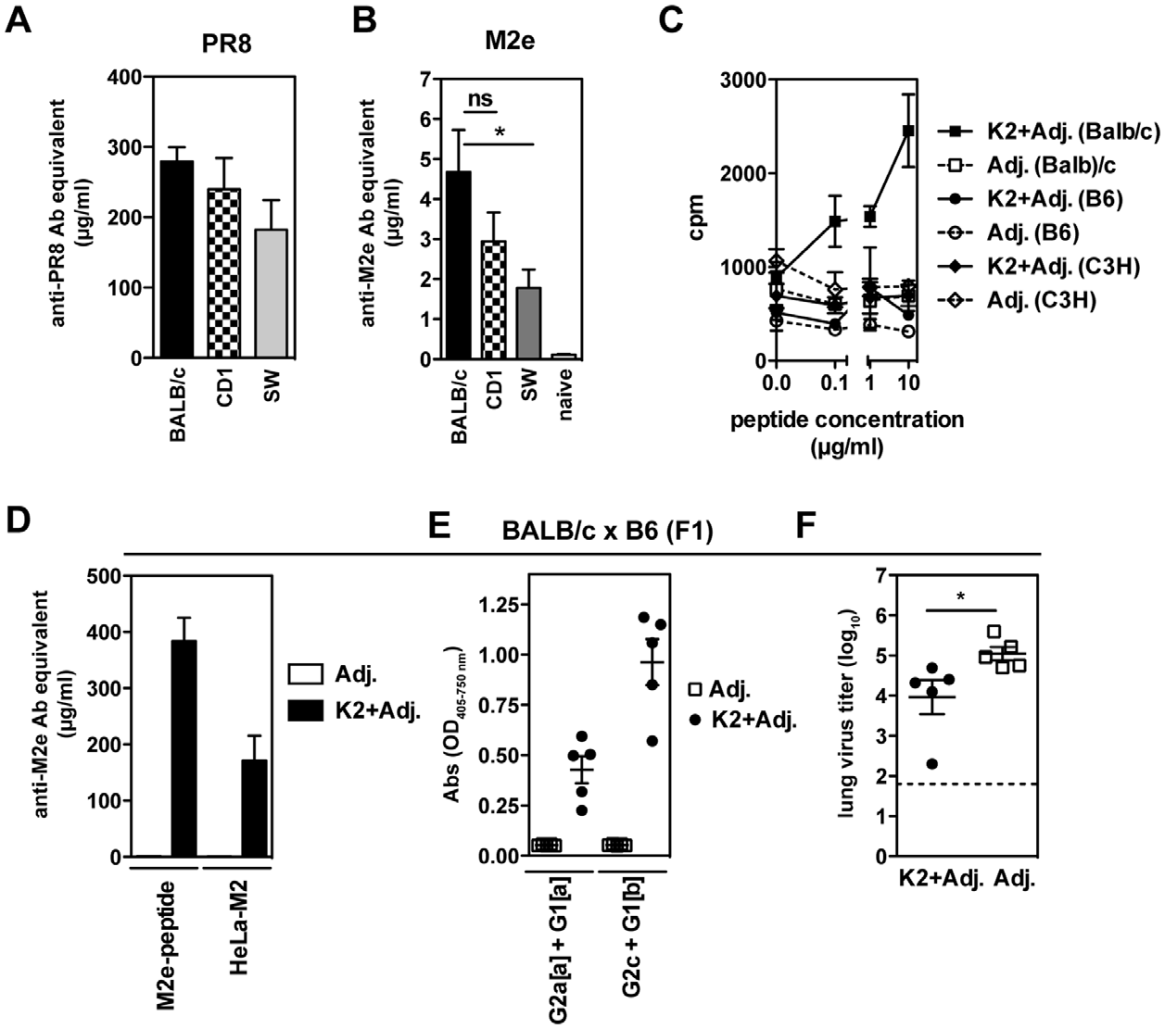
Genetic impact on anti-M2e immunity. (**A and B**) BALB/c, CD1/ICR and Swiss Webster (SW) mice (n = 5–6 mice) received three consecutive infections, first with influenza virus A viruses PR8, secondly with PR8-SEQ14 and thirdly with X31. PR8-specific IgG (**A**) and M2e-specific IgG (**B**) in sera were determined after first and third infection, respectively. The results of sera from naive mice (n = 1/mouse strain) were pooled. (**C**) BALB/c, C57BL/6, and C3H mice were immunized s.c. with K2 peptide and adjuvants or adjuvants alone. The draining lymph node was harvested on day 8 and proliferation was assessed by incorporation of ^[3]^H-thymidine during the fourth day of an *in vitro* culture in presence of K2 peptide. One out of two independent experiments shown. (**D–F**) BALB/cxC57BL/6 (F1) mice (n = 5 mice) were immunized i.n. with K2 and adjuvants or adjuvants alone. (**D**) M2e-specific Ab titers measured in an ELISA against M2e-peptide or HeLa-M2 cells in serum of BALB/cxC57BL/6 (F1) mice after third immunization. (**E**) M2e-specific serum Abs at a 1∶150 serum dilution were determined with allotype-specific reagents (IgG2a[a] and IgG1[a] for BALB/c and IgG2c and IgG1[b] for C57BL/6 origin). (**F**) After the third immunization, BALB/cxC57BL/6 (F1) mice were challenged with X31 (1000 TCID_50_/5 µL) and infectious virus in the lungs was determined 5 days post challenge.

Natural infections generate low Ab responses to M2e, however a previously reported scheme of multiple infections that has been shown to elicit small but significant levels of anti-M2e Abs in BALB/c mice [14], [15]. Adopting this strategy, BALB/c, CD1 and SW mice were infected in 3–4 weeks intervals first with PR8, then with PR8-Seq14, which evades HA-based neutralization and thirdly with X31 virus. Both outbred strains elicited a strong anti-PR8 serum IgG response similar to BALB/c mice (**Figure 5A**). All mouse strains generated detectable anti-M2e Abs in their serum after the third infection as compared to uninfected mice (**Figure 5B**), indicating that the B cell repertoire in these strains was capable of generating anti-M2e Abs.

To determine if the failure to induce anti-M2e Abs in response to M2e-MAPs was due to a lack of a T cell response in mouse strains other than BALB/c mice, an *in vitro* proliferation assay was performed. BALB/c, C57BL/6 and C3H mice were s.c. immunized with K2 and adjuvants and cells from the draining lymph nodes tested for their capacity to incorporate ^[3]^H-thymidine. Consistent with previous findings [15], BALB/c mice showed a proliferative response *in vitro* during restimulation with M2e-peptide in a dose-dependent manner, however cells from immunized C57BL/6 and C3H mice failed to proliferate (**Figure 5C**).

To further investigate the genetic restrictions of the B cell response to M2e we next immunized C57BL/6×BALB/c (F1) mice. As shown in **Figure 5D**, i.n. immunization with K2 and adjuvants elicited high anti-M2e-Ab titers against both M2e-peptide and HeLa-M2 cells. Allotype-specific reagents, Igh*a* for BALB/c and Igh*b* for C57BL/6, revealed that B cells from both strains were capable of producing anti-M2e Abs (**Figure 5E**). To examine whether the levels of anti-M2e Abs induced in vaccinated C57BL/6×BALB/c (F1) mice were sufficient to provide protection, mice were challenged with X31 virus and viral titers determined. As compared to control-immunized mice, vaccination with K2 peptide resulted in significantly reduced viral titers in the lungs (**Figure 5F**). Together, these results suggest that B cells from C57BL6 mice are able to recognize and respond to the M2e-MAP vaccination.

## Discussion

Anti-M2e Abs are poorly induced in humans by infection or current vaccines [12], [47], however several studies have shown the efficacy of anti-M2e Abs in protective anti-viral immunity. To improve anti-viral resistance, we evaluated a vaccine strategy with modified M2e-based multiple antigenic peptides. We investigated the nature and specificity of the M2e-specific B cell response using M2e-MAPs termed K2 and K3. Importantly, we assessed the ability of these M2e-MAPs to confer protection against viral challenge with homologous and M2-variant viruses and extended our studies to test the response in several inbred and outbred mouse strains.

Previously, immunization of BALB/c mice with a first-generation M2e-MAP, termed G40d, in combination with the adjuvants CpG and CT was shown to induce M2e-specific serum Abs and local immunity in the respiratory tract (RT) [14], [15]. We extended these studies using new M2e-MAPs that were generated using a simplified peptide synthesis that is more compatible with large-scale production and also incorporated promiscuous T helper epitopes that might be recognized by multiple haplotypes. Others have shown that the N-terminal 15 amino acid M2e peptide by itself is poorly immunogenic and failed to elicit a robust anti-M2e-Ab response in BALB/c mice in a prime-boost immunization [21]. We show here that immunization of BALB/c mice with K2 and K3 in combination with the adjuvants, CpG and CT, induced high M2e-specific serum Ab titers (**Figure 1B**). Of note, these Ab titers exceeded the detected levels in previous studies with G40d [14], [15], although the amount of anti-M2e Abs necessary for protection has not been determined. Anti-M2e Abs are not virus-neutralizing, but they can restrict viral replication and transmission, and passive transfer of anti-M2e monoclonal Abs in mice results in reduced viral titers, accelerated viral clearance and thus protection [5], [7], [8], [9], [10], [11]. Likewise, mice vaccinated with either K2 or K3 had significantly reduced viral titers when challenged with X31 virus and this protection extended to challenge with the more pathogenic PR8 virus (**Figure 1C and 1D**).

It has been hypothesized that the induction of local immunity at the site of virus entry, such as the mucosal surfaces of the nasal passages and the bronchial tree, is a critical determinant for protection to influenza virus infection (reviewed in [48], [49]). Work by several investigators has suggested that IgA in (upper) RT secretions in addition to IgG, the predominant isotype in the serum, plays a major role in antiviral immunity [50], [51], [52], [53]. We have compared the i.n. to s.c. route of K2 administration for their ability to induce M2e-specific IgA and IgG Abs and to promote protection. We show that the presence of M2e-specific IgA in BAL was dependent on i.n. administration of M2e-MAPs and correlated with IgA-producing cells in the RT (**Figure 2A and 2C**). On the other hand, s.c. immunization induced significantly higher Ab levels in the serum and was as efficient as i.n. immunization in inducing M2e-specific IgG in the BAL (**Figure 2A and 2B**). Our data are consistent with findings by other groups that i.n. vaccinations with live-attenuated influenza vaccine (LAIV), viral-like particles or inactivated viruses generate strong local Ab responses in the RT, in particular the induction of IgA, whereas parenteral/s.c. immunization elicits strong Ab responses in the serum but little IgA in the RT [28], [46], [54]. However, in contrast to these studies, we demonstrate that the protection of i.n. and s.c. immunized mice to challenge with X31 virus was similar (**Figure 2D**), suggesting that the absence of M2e-specific IgA in BAL after s.c. immunization can be compensated for high titers of anti-M2e IgG in BAL and serum. Our findings support recent observations in IgA^−/−^ mice that suggest that IgG is sufficient for M2-induced anti-viral immunity [26], [37].

Ideally, an influenza vaccine should induce broadly cross-reactive immunity and protection against variant viruses. We show that vaccination with K2 induced serum Abs that not only recognized the M2 sequence of PR8 used in the vaccine, but also bound efficiently to cells infected with influenza virus A/FM (**Figure 3B**), recognized M2e peptide from the pandemic H1N1 (**Figure 3D**) and provided protection to challenge with the mutant viruses P10L and P10H (**Figure 4B-D**). These results suggest that immunization with the M2e-MAPs induces Abs that might also be cross-reactive against certain types of avian viruses that have similar exchanges at amino acid position 10, which have been observed among recent H5, H7 and H9 isolates [9], [24], [25]. Our data corroborate the findings of other studies with M2e-based MAPs and contribute to the growing evidence that M2e-based vaccines are able to provide cross-reactive protection [19], [25], [26], [38], [55]. Furthermore, we demonstrate that induction of cross-reactive Abs can depend on the route of immunization in that s.c. as compared to i.n. administration induced higher levels M2e-specific Abs (**Figure 2B**) and thereby increasing the overall avidity of potentially cross-reactive anti-M2e Abs (**Figure 3D**).

Finally, we evaluated the response to K2 and K3 in other inbred and outbred mouse strains. In contrast to our findings in BALB/c mice, C57BL/6, C3H and the outbred mouse strains CD1/ICR and Swiss Webster exhibited poor anti-M2e Ab levels after vaccinations with K2 and K3 and were not protected from viral challenge (**Table 2** **, data not shown and** **Table 3**). Likewise, others have reported a genetic restriction to M2e-based vaccines [37]. Zhang et al. has demonstrated that the anti-M2e response in BALB/c mice was comprised of a set of highly restricted immunoglobulin genes usage [56]. This finding could explain in part the low Ab titers after natural infection as well as the unresponsiveness we observed in other mouse strains. However, we demonstrate that multiple infections in outbred mice induced a small but detectable serum Ab response to M2e (**Figure 5B**). Moreover, M2e-specific Abs from both BALB/c and C57BL/6 allotypes were detected in vaccinated BALBxB6 F1 mice (**Figure 5E**), indicating that the lack of an Ab response to M2e-MAPs in C57Bl/6 mice was not due to an inherent failure of B cells from this background to recognize M2e.

A key feature of the modified M2e-MAPs used in this study is that they contain potentially universal T cell determinants. The T cell epitopes included in K2 and K3 vaccine constructs have previously been tested for promiscuity and were shown to bind many HLA-DR molecules [44], [45], [57]. While efficacy in inducing T cell responses is variable and shows some genetic dependence, other studies demonstrated that the T cell epitope CS_327–345_ induces responses in C57BL/6 mice and non-human primates as a linear or branched peptide together with water-in-oil based adjuvants or CpG [58], [59]. Likewise, a MAP-combination of CS_381–396_ with the B cell epitope (NANP)_3_ induces responses in multiple inbred mouse strains [57]. Additionally, polypeptide constructs with multiple T cell epitopes, including HA_307–319_, HbsAg_19–33_, and CS_381–396_ conjugated to *Haemophilus influenzae* polysaccharide elicit Ab titers in CD1 outbred mice [43]. However, we were unable to detect T cell responses in C57BL/6 and C3H mice after s.c. immunization with K2 and adjuvants (**Figure 5C**). Thus, the lack of a T cell response to the immunizing M2e-MAP peptide could explain the lack of measurable M2e-specific Abs.

A fully synthetic peptide vaccine based on a highly conserved viral antigen has the advantage that its preparation does not include undesirable components from the purification processes of live whole organisms and that it does not require yearly adjustments. However, the main question that still needs to be addressed for this vaccine approach is how to improve its immunogenicity. Although we have not formerly shown that the adjuvants used in this study, CpG and CT, have comparable effects in different mouse strains, studies by others suggest that their adjuvant activity is independent of genetic backgrounds [60], [61]. Embedding additional adjuvant sequences into MAPs as well as incorporating other conserved immunogenic viral target proteins, such as NP and HA, for a combination therapy have been shown to exceed protective efficacy compared to single antigens and may enhance their therapeutic potential [29]. Rapid manufacturing of an influenza vaccine based on M2e-containing MAP constructs provided with optimal T cell help may be an attractive alternative for the production of current influenza vaccines.

## Materials and Methods

### Ethics Statement

All animal studies were performed under the approved protocol #112046 issued by the Wistar Institutional Animal Care and User Committee.

### Mice

Female mice, including the inbred mouse strains BALB/c, C57BL/6, C3H and BALB/cxC57BL/6 (F1) mice and outbred mouse strains, CD1/ICR and Swiss Webster, 8–10 weeks old, were purchased from the National Cancer Institute and Charles River laboratories. All mice were housed at the Wistar Institute under specific pathogen free/viral antibody free conditions.

### Peptides

Multi-antigenic peptide constructs, designated K2 and K3 were synthesized containing 4 side chains of M2e (SLLTEVETPIRNEWG) and 2 pairs of T helper determinants from the circumsporozoite protein from *Plasmodium falciparum* (CS_381–396_ KKIAKMEKASSVFNVV and CS_327–345_ YLNKIQNSLSTEWSPCSVT), or the hepatitis B virus antigen (HbsAg_19–33_ FFLLTRILTIPQSLD and the influenza virus hemagglutinin (HA_307–319_ PKYVKQNTLKLAT) and are depicted in **Table 1**.

The linear M2e peptide from the pandemic influenza virus A/Texas/05/2009 (H1N1) was synthesized based on the sequence according to GenBank Accession number FJ966968.1.

Peptides with M2e-sequence from escape mutants of influenza virus PR8, termed P10H and P10L were described previously [9].

### Immunization

The immunization protocol was adopted from Mozdzanowska et al. [14], [15]. In brief, 50 µL sterile PBS containing 5 µg of M2e-MAPs K2 or K3 (see **Table 1**) together with the adjuvants CpG ODN 1826 (3 µg, Sigma) and cholera toxin (CT, 0.5 µg, Sigma) or adjuvants alone were administered intranasally (i.n.) or subcutaneously (s.c., into the tail base) into anesthetized mice (ketamine/xylazine, 70/7 mg/kg). Vaccinations were repeated twice in 3–4 weeks intervals.

### Sample collections and tissue preparation

Prior to each vaccination, blood samples were collected from each immunization group by mandibular bleeding or obtained from the tail vein. Tissues were collected and cells prepared as described [62]. In brief, mice were euthanized by CO_2_ asphyxation and blood was collected through cardiac puncture. BAL was harvested by flushing the airway compartment with 0.8 mL PBS with 1% FBS (PBS/FBS) 3 times. Perfused lungs were digested in HBSS with 400 U/mL Collagenase D (Roche) for 30 min at 37°C. Cells from bone marrow were prepared from 2 hind legs of mice. Tissues were passed through metal wire mesh and after red blood cell lysis, cells resuspended in Iscove's complete medium containing 10% FBS for ELISPOT assays.

For viral titers (see below), noses and lungs dissected after exsanguinating mice and tissues were stored at −80°C until processed for virus titration.

### Viruses and infection

The following viruses were used in this study: A/PR/8/34 (PR8, H1N1), A/HKx31 (X31, H3N2), A/FM1/49 (FM, H1N1) and influenza B/Lee/40 (B/Lee). The virus escape mutants P10H and P10L are viral isolates that emerged from PR8-infected SCID mice, which had been chronically treated with M2e-specific Ab 14C2 and have been described elsewhere [9]. For challenge experiments, a viral dose of 1000 TCID_50_ was administered i.n. into anesthetized mice (ketamine/xylazine, 70/7 mg/kg) in 5 µL or 50 µL sterile PBS as indicated.

### Virus titer

To determine protection of vaccinated mice, titers of infectious virus were measured in noses and lungs 4 and 5 days after viral challenge as described previously [63], [64]. Tissues were homogenized in sterilized mortars and transferred to a tube, and centrifuged for 5 min at 400×g (1300 rpm). The supernatant was tested for concentration of infectious virus in the Madin-Darby canine kidney cells (MDCK) assay [64]. Lung titers are expressed as dilution of lung extract at which 50% of the MDCK cultures revealed virus growth (TCID_50_/mL).

### *In vitro* infection of MDCK cells

Influenza strains PR8, FM, B/Lee, P10H and P10L were used for *in vitro* infection of MDCK cultures as described [9]. In brief, MDCK cells (ATCC) were grown to form confluent monolayers in flat-bottom 96-well Falcon Microtest plates (Becton Dickinson). Monolayers were infected by incubation (90 min at 37°C) with 50 µL of virus (∼10^6^ TCID_50_). Typically, eight replicate cultures were infected with a given dilution of a virus isolate. After 90 min, 100 µL of Iscove's media, 5% FCS was added to each well, and incubation was continued as described above for another 7 to 8 h. Monolayers were then washed with PBS, fixed by incubation with 5% buffered formalin (Fisher Scientific) for 5 min at room temperature, washed again with PBS, and blocked and stored with PBS, 1% BSA at 4°C. The infected MDCK cell monolayers were then tested for reaction with immune sera from vaccinated mice or M2e-specific monoclonal Ab (1 to 2 µg/mL) as standard as described in the ELISA protocol below.

### ELISA and ELISPOT assay

Measurement of M2e-specific Antibodies by ELISA was performed as described [12], [14], [15]. In brief, two approaches were taken to measure M2e-specific Ab by ELISA; immunosorbents cys-M2e peptide and control peptide (cys-backbone) or HeLa cells stably transfected with M2 (HeLa-M2) or empty control plasmid (HeLa-C10) were used to distinguish between specific and non-specific binding [12], [14], [15]. HeLa-M2 and HeLa-C10 cells were obtained from Dr. Gerhard (The Wistar Institute). Each assay was standardized and quantified by titration of purified M2e-specific mAb 14C2-S1-4 (G2a) on the same immunosorbents and Ab titers in test samples were defined as anti-M2e Ab equivalent. PR8-specific Abs were standardized based on purified anti-HA Ab standard H36-4-5.2 (G2a). Abs in serum or BAL samples were detected with biotinylated mAb 187 and subsequent avidine-coupled to alkaline phosphatase (AP) or anti-mouse IgG- or IgA-AP and developed with pNPP as described (Sigma). The difference in optical density (OD) between specific and non-specific immunosorbent was used for quantification of Ab concentration. ELISA data were collected with an E-max ELISA reader and analyzed with Softmax Pro software (Molecular Devices).

To detect M2e-specific ASCs, the same immunosorbents were used to coat ELISPOT plates (Millipore). Cell suspensions from various organs were titrated and incubated for 6–7 h at 37°C in a humidified incubator with 5% CO_2_. Immunosorbent-bound Abs were detected with anti-mouse IgG- or IgA-AP and developed with NBT/BCIP substrate (Sigma). Automated spot counts were performed with an ImmunoSpot Reader (CTL) and Immunospot satellite software (CTL).

### Proliferation assay

Mice were immunized s.c. with 50 µL sterile PBS containing 10 µg of K2 peptide together with adjuvants or adjuvants alone into both flank sides. Eight days following immunization, mice were euthanized and draining inguinal lymph nodes as well as spleens harvested. For preparation of antigen presenting cells, spleens were processed into single cell suspensions as described, resuspended at 5×10^6^/mL in Iscove's media, 10% FBS and irradiated with 2000 rad using a cesium source. 0.5×10^6^ responder cells from the inguinal lymph node were cultured in a 96 well plate with an equal number of irradiated splenocytes in the presence of indicated concentrations of K2 peptide. Three days later, ^[a]^H-thymidine was added (1 µCi/well) and plates harvested after 24 h as described [15].

### Statistics

Data are presented as mean ± standard error of the mean (SEM). Statistical significance between two groups was calculated using the unpaired Student's t-test. For three and more groups, one-way ANOVA with multiple comparison post-test was used. Statistical significance with data below detection limit was calculated using a two-part t-test or Fisher's exact test. Statistical tests were performed using Prism software (GraphPad Software). P-values are depicted as follows: ns (not significant), p>0.05; *, p<0.05; **, p<0.01 and ***, p<0.001.
